# USING PHOTOVOICE TO UNDERSTAND SOCIAL MODEL HOSPICE FROM RESIDENTS’ PERSPECTIVES

**DOI:** 10.1093/geroni/igad104.1195

**Published:** 2023-12-21

**Authors:** Francine Jensen

**Affiliations:** Utah Valley University, Orem, Utah, United States

## Abstract

Individuals who experience homelessness and are diagnosed with six months or less to live are disadvantaged at a critical time of life. Many people choose to die at home using hospice services supported by family or close friends, but those without stable housing are unable to access hospice, demonstrating inequitable access at the end of life. For some, social model hospice (SMH) care is being provided free of charge by communities to increase access to hospice services for these individuals. The literature about SMH is growing, but currently missing are the voices of those receiving this care and its impacts on their end-of-life experiences. Using photovoice, a community-based participatory method, three residents shared their first-person perspectives using disposable cameras to document meaningful aspects of care, both positive and negative, and helped researchers analyze themes. Until this study, photovoice had not been used with people experiencing homelessness at the end of life. We found participants eager to take part in all aspects of photovoice as their health permitted. Their perspectives documented and informed the humanistic side of SMH care, illuminating the value of respect, care, autonomy, meeting physical needs, and forming supportive relationships; few criticisms were offered. Consistent with photovoice aims, this method is a powerful research tool involving a disadvantaged group who provided visual commentary on what is working and what could improve at one SMH location. Findings can inform policy decisions for communities interested in starting SMHs and help functioning SMHs make programmatic decisions to support vulnerable residents.

